# Satralizumab as an add-on treatment in refractory pediatric AQP4-antibody-positive neuromyelitis optica spectrum disorder: a case report

**DOI:** 10.3389/fimmu.2023.1257955

**Published:** 2023-10-17

**Authors:** Xiaojing Li, Wenlin Wu, Yiru Zeng, Wenxiao Wu, Chi Hou, Haixia Zhu, Yinting Liao, Yang Tian, Zongzong Chen, Bingwei Peng, Wen-Xiong Chen

**Affiliations:** Department of Neurology, Guangzhou Women and Children’s Medical Center, Guangzhou Medical University, Guangzhou, Guangdong, China

**Keywords:** pediatric, NMOSD, AQP4, relapse, satralizumab

## Abstract

Neuromyelitis optica spectrum disorder (NMOSD) is a rare autoimmune disease of the central nervous system. Relapse and incomplete recovery from relapse are common in NMOSD. Most patients with NMOSD have IgG to aquaporin-4 (AQP4-IgG). New biological agents for AQP4-IgG-seropositive NMOSD, such as satralizumab, have become available for maintenance therapy. Satralizumab is an anti-interleukin-6 receptor monoclonal antibody. To date, few studies have evaluated satralizumab as an add-on treatment in pediatric NMOSD patients. Here, we report an 11-year-old girl with NMOSD who frequently relapsed under long-term treatment, including oral prednisone, rituximab, mycophenolate mofetil (MMF), and maintenance intravenous immunoglobulin treatment even with B-cell depletion. For the poor treatment response and to improve the efficacy of relapse prevention further, the patient received satralizumab treatment as an add-on therapy to MMF plus oral prednisone, with a dose of 120 mg administered subcutaneously at weeks 0, 2, and 4 and every 4 weeks after that. After initiating satralizumab, the patient remained relapse-free for 14 months at the last follow-up. Satralizumab might be effective and safe as an add-on treatment in refractory pediatric AQP4-IgG-seropositive NMOSD under B-cell depletion.

## 1 Introduction

Neuromyelitis optica spectrum disorder (NMOSD) is a rare autoimmune disease of the central nervous system, characterized by inflammatory lesions mainly affecting the optic nerve, spinal cord, brainstem, and cerebrum (1). Around two-thirds or more of patients with NMOSD have IgG to aquaporin-4 (AQP4-IgG) (2). AQP4-IgG-seropositive NMOSD is more common in adults than children, with female preponderance (1). More than 80% of patients experience relapses after a median follow-up of 58 months (3). Incomplete recovery from relapse is common, leading to the accrual of permanent, residual disability. In one study of European patients with AQP4-IgG-seropositive NMOSD, a median Expanded Disability Status Scale was 5 at the last follow-up examination (median disease duration 60 months, range 0–390 months) (3). Disease management of NMOSD focuses on treating acute attacks to improve recovery and long-term treatment to prevent relapse.

For NMOSD, the initial treatment of acute attacks consists of intravenous methylprednisolone (IVMP), whereas escalatory or rescue treatments for patients who fail to recover substantially with IVMP include plasma exchange, immunoadsorption (IA), and intravenous immunoglobulin (IVIG) (1). The long-term treatment includes azathioprine, rituximab (RTX), mycophenolate mofetil (MMF), tocilizumab, and maintenance IVIG (1). Recently, new biological agents for AQP4-IgG-seropositive NMOSD, such as satralizumab, have become available for maintenance therapy. Satralizumab is an anti-interleukin-6 receptor monoclonal antibody (2). To date, few data have been available for pediatric patients successfully treated with satralizumab (2). We report satralizumab as an add-on therapy in a pediatric NMOSD patient who frequently relapsed under long-term treatment, including oral prednisone, RTX, MMF, and maintenance IVIG.

## 2 Case description

**Figure 1:**
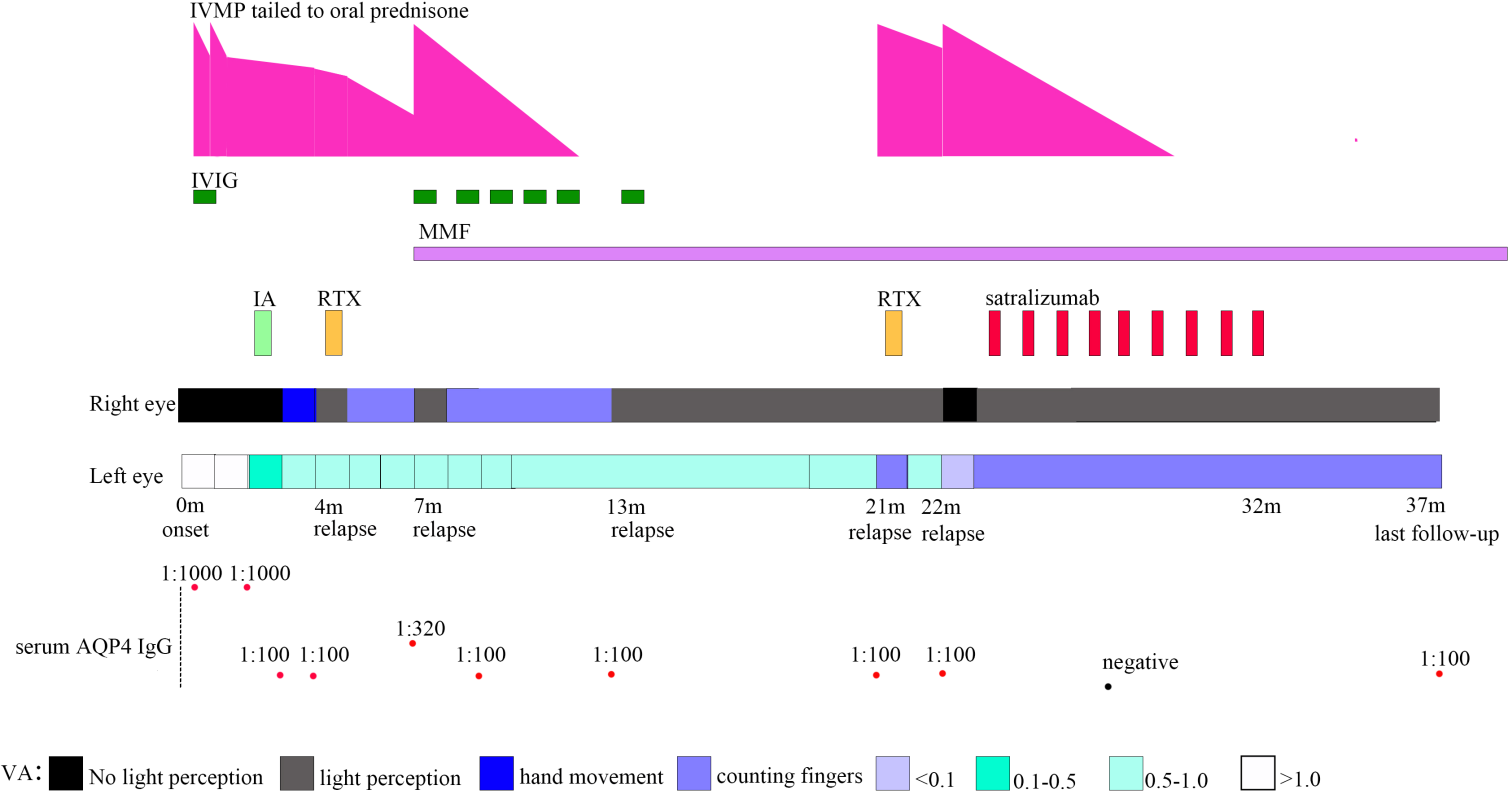
Timeline depicting visual acuity change, immunotherapy, and serum AQP4 IgG. VA, visual acuity; AQP4, aquaporin-4; IA, immunoadsorption; IVIG, intravenous immunoglobulin; IVMP, intravenous methylprednisolone; MMF, mycophenolate mofetil; RTX, rituximab.

**Figure 2:**
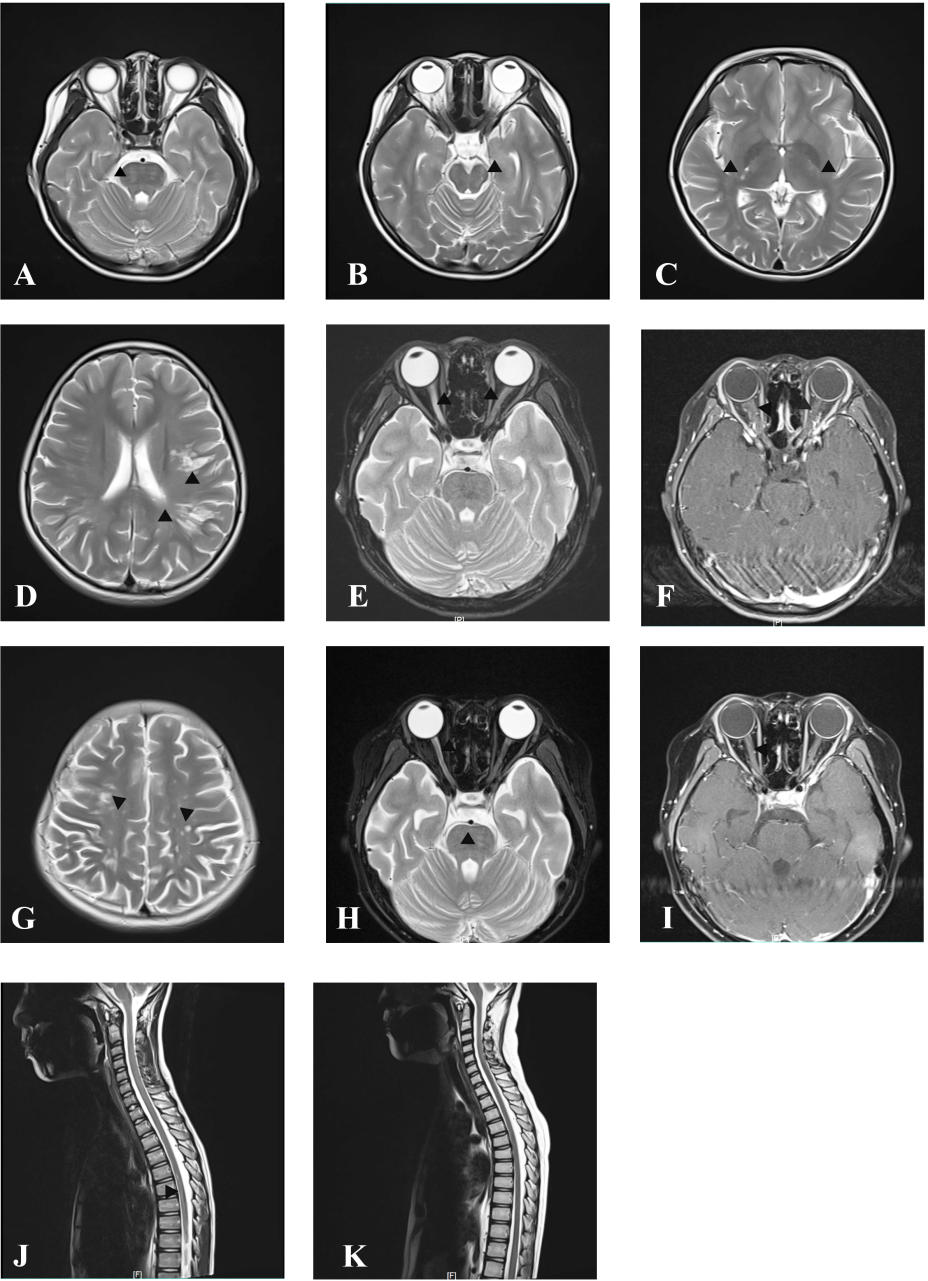
MRI at onset and follow-up. Axial cranial T2-weighted images showing T2-hyperintense lesions in pons **(A)**, midbrain **(B)**, posterior limb of internal capsule **(C)**, and subcortical white matter of the frontal and parietal lobe **(D)**. Axial orbital T2-weighted image showing longitudinally extensive T2-hyperintense lesions in the bilateral optic nerve **(E)** with gadolinium enhancement in the T1-weighted gadolinium-enhanced image **(F)**. Axial cranial T2-weighted images showing new T2-hyperintense lesions in subcortical and deep white matter of the frontal, parietal, and occipital lobes after treatment with IVMP and IVIG at onset attack **(G)**. Axial orbital T2-weighted image showing that lesions in the left optic nerve disappeared, whereas in the right optic nerve they became smaller without gadolinium enhancement in T1-weighted gadolinium-enhanced image at follow-up after immunosorbent therapy **(H, I)**. Sagittal spinal T2 weighted image showing T2-hyperintense lesions at onset **(J)** and resolution of lesions at follow-up after immunosorbent therapy **(K)**.

A previously healthy 11-year-old girl presented with rapidly progressive visual impairment in her right eye for 10 days in March 2020 and was admitted to our hospital. Neurological examination on admission showed that the eye pupil diameter was 5 mm in the right and 3 mm in the left. Pupil examination showed a right relative afferent pupillary defect. Snellen visual acuity (VA) test showed that the right eye was blind without any light perception, and VA on the left was 1.5 (**Figure 1**). The patient’s cerebrospinal fluid (CSF) showed a normal white cell count and protein. CSF tested negative for the nucleic acid of enterovirus, influenza A, influenza B, herpes simplex virus, Epstein–Barr virus, and cytomegalovirus. Oligoclonal bands in CSF and serum were negative. The results of the AQP4 antibody test by cell-based assay were positive in both serum (titer, 1:1,000) and CSF (titer, 1:3.2). Cytokine test showed that interleukin-6 (IL-6) (136.05 pg/ml in CSF, 58.14 pg/ml in the serum, normal reference range: 0–8.88 pg/ml) and IL-8 (305.47 pg/ml in CSF, 26.13 pg/ml in serum, the normal reference range 0–15.71 pg/ml) in both CSF and serum increased, and IL-2 (28.21 pg/ml, normal reference range: 0–5.03 pg/ml) in the serum increased. The visual evoked potential exhibited bilateral reduced P100 amplitudes with normal latency, with more severity in the right. Brain magnetic resonance imaging (MRI) results showed T2-hyperintense lesions in the pons, midbrain, posterior limb of the internal capsule, and subcortical white matter of the frontal and parietal lobe without gadolinium enhancement (**Figures 2A–D**). The orbital MRI showed a longitudinally extensive T2-hyperintense lesion with gadolinium enhancement in T1-weighted gadolinium-enhanced imaging in the bilateral optic nerves (**Figures 2E, F**). The spinal MRI showed a T2-hyperintense lesion from T5 to T6 without gadolinium enhancement (**Figure 2J**). The patient was treated with two courses of IVMP (15 mg/kg/day for 3 days tailed to oral prednisone 40 mg/day) in combination with IVIG (total 2 g/kg split over 3 days). Moreover, there was no improvement in visual impairment. Furthermore, VA on the left was reduced to 0.25, together with new T2-hyperintense lesions found in the subcortical and deep white matter of the frontal, parietal, and occipital lobes and bilateral thalamus (**Figure 2G**). Then, protein A IA was started. She received three sessions of IA on each alternative day. After treatment of IA, VA on the left was improved to 0.6, and on the right it was improved to “hand movement” from 70 cm (**Figure 1**). MRI showed that T2-hyperintense lesions in the spinal cord and left optic nerve disappeared, in the right optic nerve it became smaller, and in the brain it was similar to previous results (**Figures 2H, I, K**). Serum AQP4 antibody titer decreased to 1:100. Then, the patient was discharged and continued to take oral prednisone. After discharge, the patient underwent an optical coherence tomography examination in an ophthalmology hospital. The optical coherence tomography results showed thinning of the retinal nerve fiber layer (57 μm on the right, 83 μm on the left) and the ganglion cell layer (50 μm on the right, 67 μm on the left).

One month after IA in July 2020, the patient’s VA on the right was reduced to only light perception (**Figure 1**). RTX administration (375 mg/m² of the body surface area weekly for 4 consecutive weeks) was started. After RTX treatment, the patient’s VA on the right was improved to “counting fingers” from 30 cm. The percentage of CD19^+^ B cells in periphery blood lymphocytes decreased to 0.04%, and the periphery blood CD20^+^ B-cell count decreased to 1 cell/μl. Serum AQP4 antibody titer was the same as the previous one (1:100).

Two months after RTX treatment in October 2020, the patient relapsed with right eye blindness without light perception under prednisone treatment, tailed to 12.5 mg/day (**Figure 1**). The blood CD19^+^ B cells were still depleted. Serum AQP4 antibody titer increased to 1:320. MRI showed no new lesions. The patient received IVMP treatment (15 mg/kg/day for 3 days, tailed to oral prednisone 40 mg/d) in combination with IVIG (2 g/kg split over 5 days). After treatment with IVMP and IVIG, the VA on the right was improved to light perception. Then, MMF (initial 250 mg/day) treatment was started as a long-term treatment to reduce relapse. Four months after MMF initiation, the patient received monthly IVIG treatment (400 mg/kg/day for 1 day monthly).

**Figure 3:**
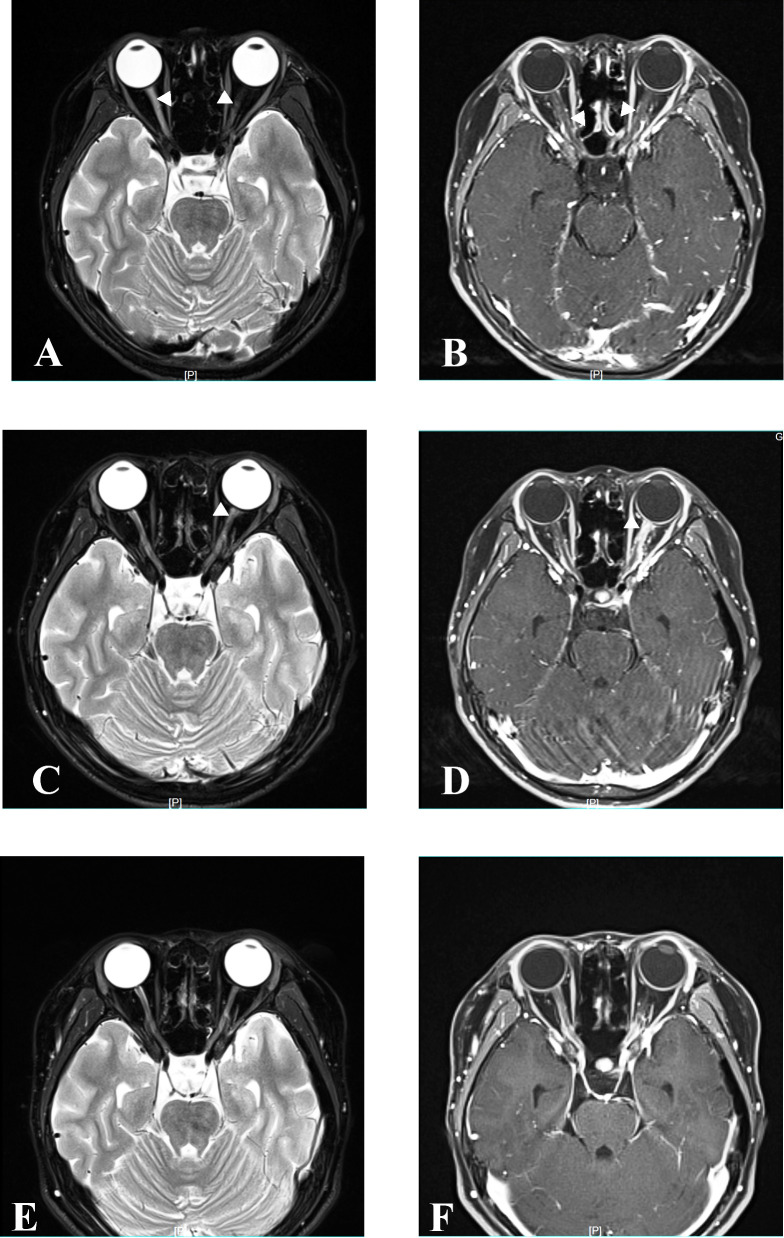
MRI at relapse and follow-up. Axial orbital T2-weighted image showing lesions in bilateral optic nerve with gadolinium enhancement in the T1-weighted gadolinium-enhanced image at relapse in April 2021 **(A, B)**. Axial orbital T2-weighted image showing lesions in the left optic nerve with gadolinium enhancement in the T1-weighted gadolinium-enhanced image at relapse in December 2021 **(C, D)**. Axial orbital T2-weighted image showing that lesions disappeared in the bilateral optic nerve at the last follow-up in April 2023 **(E, F)**.

Six months after MMF treatment in April 2021 (around 1 month after prednisone withdrawal), the patient relapsed with VA on the right reduced to light perception (**Figure 1**) and MRI showed new T2-hyperintense lesions in the bilateral optic nerve with gadolinium enhancement in T1-weighted gadolinium-enhanced image (**Figures 3A, B**). Serum AQP4 antibody titer was the same as the previous (1:100). The patient received IVIG treatment (400 mg/kg), and the dose of MMF increased from 500 to 750 mg/day.

Eight months later, in December 2021, the patient relapsed with VA on the left reduced to “counting fingers” from 20 cm (**Figure 1**). Serum AQP4 antibody titer was the same as the previous (1:100). One week before relapse, the patient had an exposure history of varicella. Serum IgM of the varicella-zoster virus was negative, whereas serum IgG of the varicella-zoster virus was positive. Blood next-generation sequencing for bacteria, viruses, fungi, and parasites with known genome sequences showed two sequences for the human herpes virus 7 and one for cytomegalovirus. MRI showed new T2-hyperintense lesions in the left optic nerve with gadolinium enhancement in the T1-weighted gadolinium-enhanced image (**Figures 3C, D**). The patient received acyclovir treatment, IVMP treatment (20 mg/kg/day for 3 days tailed to oral prednisone 40 mg/day), and RTX treatment. However anaphylactic reaction occurred during the first week of RTX treatment, and RTX treatment was discontinued, and the patient received IVIG treatment (400 mg/kg). After this series of treatments, the patient’s VA on the left was improved to 0.8.

One month later, in January 2022, the patient relapsed with the VA on the right reduced to blindness without light perception, and on the left reduced to 0.05 under the blood CD19^+^ B-cell depletion (**Figure 1**). The serum AQP4 antibody titer was the same as the previous one (1:100). MRI showed that previous T2-hyperintense lesions in the left optic nerve disappeared. The visual evoked potential showed bilateral prolonged P100 latency with severely reduced amplitudes. The patient received IVMP treatment (20 mg/kg/day for 3 days, tail to oral prednisone 40 mg/day). After IVMP was tailed to oral prednisone, the VA on the right was not improved and the VA on the left was reduced “counting fingers”. For the poor treatment response and to improve the efficacy of relapse prevention further, satralizumab was started in February 2022, with a dose of 120 mg administered subcutaneously at weeks 0, 2, and 4 and every 4 weeks after that. One month after the initial satralizumab, VA on the right was improved to light perception, and satralizumab could be administered by the patient’s parents. No hypersensitivity reactions or adverse events such as infections, blood cell abnormalities, or liver dysfunction were noted during the satralizumab treatment. Serum AQP4 antibody turned negative 4 months after satralizumab initiation. Oral prednisone (35 mg/day) was tapered off 5 months after satralizumab initiation. In December 2022, the patient gave up satralizumab treatment for unaffordable satralizumab and continually received MMF (750 mg/day) treatment.

At the latest clinical follow-up in April 2023, the patient remained relapse-free for 14 months after initiating satralizumab. VA on the right was light perception, and on the left was “counting fingers” from 60 cm (**Figure 1**). The serum AQP4 antibody titer was 1:100. No new lesions were found in the brain and orbital MRI (**Figures 3E, F**).

## 3 Discussion

Here, we reported a pediatric NMOSD patient who was AQP4 antibody positive treated with satralizumab. *In vivo* and *in vitro* experiments indicate that AQP4-IgG is pathogenic in NMOSD. AQP4-IgG, targeting AQP4 highly expressed in the astrocytic end-feet, belongs to the IgG subclass 1 and can activate the complement system, leading to lysis and cell death (1). Activated B and T cells, innate immunity cells, and pro-inflammatory cytokines play roles in the pathophysiology of NMOSD (4). Immunotherapies, including IVMP, plasma exchange, and IVIG, are commonly used in acute attacks. Azathioprine and MMF are frequently used for relapse prevention. With the development of the understanding of the pathophysiology of NMOSD, several monoclonal antibodies, including RTX, eculizumab, inebilizumab, satralizumab, tocilizumab, bevacizumab, and ublituximab, were applied in clinical trials to assess the effect on preventing relapse (5).

RTX was first used monoclonal antibody empirically in NMOSD to prevent relapse and is typically administered as induction therapy (375 mg/m^2^ of body surface area weekly for 4 consecutive weeks, or 1 g/week infused twice at 2-week intervals) followed by maintenance therapy on various schedules (6, 7). RTX effectively reduces the mean annualized relapse rate and Expand Disability Status Scale score in NMOSD patients, but some patients still experience relapse even under B-cell depletion (5). For our patient, RTX was introduced at the first relapse. However, the patient relapsed under B-cell depletion only 2 months after RTX. The AQP4 antibody in NMOSD was secreted by CD19^int^CD27^high^CD38^high^CD18^−^ plasmablasts in an IL-6-dependent manner (8). Moreover, CD19^int^CD27^high^CD38^high^CD18^−^ plasmablasts do not express CD20 and cannot be depleted by RTX, causing resistance to RTX treatment. In addition, infusion-related adverse events (AEs) were the most common AEs in RTX treatment for NMOSD (6). Our patient had an anaphylactic reaction with the second course of RTX treatment and had to discontinue RTX.

The switch from RTX therapy to another treatment regimen in refractory NMOSD, especially for pediatric patients, is debatable. A meta-analysis of four RCTs containing 524 patients showed no significant differences in lowering the relapse risk between B-cell-targeted monoclonal antibodies, including RTX and inebilizumab, and other monoclonal antibodies including eculizumab and satralizumab (5). Inebilizumab and eculizumab were approved for patients older than 18 years (9, 10).

Satralizumab is a humanized monoclonal antibody targeting membrane-bound and soluble IL-6 receptors. IL-6 levels increased significantly in patients with NMOSD during relapse and correlated with relapse severity (11). IL-6 is thought to play an important role in NMOSD pathophysiology. IL-6 signaling stimulates B-cell differentiation and promotes AQP4 antibody secretion. IL-6 can disturb the generation of FOXP3^+^ regulatory T cells and promote proinflammatory T-helper subtype 17 (TH17) cells (6, 12). TH17 cells, along with IL-6, can promote the differentiation of B cells into AQP4-IgG–producing plasmablasts (6). Furthermore, IL-6 can increase the permeability of the brain–blood barrier (1, 5). The phase-3 SAkuraSky study assessed the efficacy and safety of subcutaneous satralizumab as an add-on therapy to oral immunosuppressive drugs in NMOSD patients aged 12–74 years. Among the AQP4-IgG-seropositive NMOSD, relapse occurred in 11% of those in the satralizumab group and 43% in the placebo group (hazard ratio, 0.21; 95% CI, 0.06 to 0.75) (2). Moreover, the SAkuraStar phase-3 study compared satralizumab monotherapy with placebo for adult patients with NMOSD and found that satralizumab monotherapy effectively lowered the risk of relapse more than the placebo (13). Satralizumab was introduced to our patient as an add-on therapy to MMF plus oral prednisone. The patient had remained relapse-free for 14 months after initiating satralizumab at the last follow-up. The annualized relapse rate before satralizumab treatment was 2.2/person-year with an observation period of 21 months, which reduced to 0 after satralizumab treatment with an observation period of 14 months. No hypersensitivity reactions or adverse events occurred during satralizumab treatment. The patient gave up satralizumab treatment for unaffordable satralizumab. In China, the Chinese Health System does not cover satralizumab treatment. The patient may relapse again without the effect of this anti-interleukin-6. Tocilizumab is another humanized anti-interleukin-6 receptor monoclonal antibody used more commonly in adult rheumatoid arthritis, juvenile idiopathic arthritis, and cytokine release syndrome patients aged ≥2 years. Unlike satralizumab with class 1 evidence for use in AQP4-IgG-seropositive NMOSD, several retrospective case series and reports suggested the efficacy of tocilizumab in the treatment of NMOSD even in patients who had failed B-cell therapies (14). Tocilizumab is much cheaper than satralizumab in China. Satralizumab was designed using recycling antibody technology™ to last longer in circulation and, thus, have a longer half-life than tocilizumab (14). It is unknown whether there is a difference in the effects between satralizumab and tocilizumab treatment on NMOSD. For our patient, tocilizumab may be a choice for long-term treatment to prevent relapse.

## 4 Conclusions

The present case suggests that satralizumab might be effective and safe as an add-on treatment in refractory pediatric AQP4-IgG-seropositive NMOSD under B-cell depletion. More cases need to be evaluated.

## Data availability statement

The original contributions presented in the study are included in the article/supplementary material. Further inquiries can be directed to the corresponding authors.

## Ethics statement

The studies involving humans were approved by the Ethics Committee of Guangzhou Women and Children Medical Center (Approval No: [2019]40701). The studies were conducted in accordance with the local legislation and institutional requirements. Written informed consent for participation in this study was provided by the participants’ legal guardians/next of kin. Written informed consent was obtained from the individual(s), and minor(s)’ legal guardian/next of kin, for the publication of any potentially identifiable images or data included in this article.

## Author contributions

XL: Conceptualization, Funding acquisition, Investigation, Writing – original draft, Writing – review & editing. WLW: Conceptualization, Funding acquisition, Investigation, Writing – original draft, Writing – review & editing, Data curation. YZ: Conceptualization, Data curation, Investigation, Writing – original draft. WXW: Conceptualization, Data curation, Investigation, Writing – original draft. CH: Conceptualization, Investigation, Writing – review & editing. HZ: Conceptualization, Data curation, Investigation, Writing – review & editing. YL: Conceptualization, Data curation, Writing – review & editing. YT: Data curation, Investigation, Writing – review & editing. ZC: Data curation, Formal Analysis, Writing – review & editing. BP: Data curation, Formal Analysis, Visualization, Writing – review & editing. W-XC: Conceptualization, Investigation, Project administration, Visualization, Writing – review & editing.
